# Exploring factors influencing the compliance of patients and family carers with infection prevention and control recommendations across Bangladesh, Indonesia, and South Korea

**DOI:** 10.3389/fpubh.2022.1056610

**Published:** 2022-12-22

**Authors:** Ji Yeon Park, Jerico Franciscus Pardosi, Titik Respati, Eka Nurhayati, Md. Saiful Islam, Kamal Ibne Amin Chowdhury, Holly Seale

**Affiliations:** ^1^School of Population Health, University of New South Wales, Sydney, NSW, Australia; ^2^School of Public Health and Social Work, Queensland University of Technology, Brisbane, QLD, Australia; ^3^Faculty of Medicine, Universitas Islam Bandung, Bandung, West Java, Indonesia; ^4^Infectious Diseases Division, International Centre for Diarrhoeal Disease Research, Dhaka, Bangladesh

**Keywords:** healthcare-associated infection, infection control, caregiver, hand hygiene, Bangladesh, Indonesia, Korea (Rep.), communication

## Abstract

**Background:**

Poor compliance with infection prevention and control (IPC) measures has been a longstanding issue globally. To date, healthcare workers (HCWs) have been the primary target for policy and strategy revisions. Recent studies exploring the contributing factors to the spread of COVID-19 across countries in Asia have suggested that the scope of focus should be extended to family carers who provide patient care activities. This study aimed to explore factors affecting patients' and their family carers' IPC compliance in hospitals in Bangladesh, Indonesia, and South Korea.

**Method:**

A qualitative study incorporating 57 semi-structured interviews was conducted in five tertiary-level hospitals across the three focus countries between July 2019 and February 2020. Interviews were undertaken with: (1) patients, family carers and private carers; and (2) healthcare workers, including nurses, doctors, and hospital managers. Drawing upon the principles of grounded theory, data were inductively analyzed using thematic analysis.

**Results:**

A total of three main themes and eight subthemes are identified. Key themes focused on the assumptions made by healthcare workers regarding the family/private carers' level of understanding about IPC and training received; uncertainty and miscommunication regarding the roles of family/private carers; variations in carer knowledge toward IPC and healthcare-associated infections, and the impact of cultural values and social norms.

**Conclusion:**

This exploratory study offers novel findings regarding the factors influencing IPC compliance among patients and their family/private carers across various cultural settings, irrespective of resource availability. The role of cultural values and social norms and their impact on IPC compliance must be acknowledged when updating or revising IPC policies and guidelines.

## Introduction

Healthcare-associated infections (HAI) strain healthcare systems by increasing patients' morbidity and mortality. Crude estimates from the World Health Organization (WHO) suggest that 1 in 10 patients admitted to hospitals have died from HAI (1). More specifically, approximately 7 in 100 patients hospitalized in high-resource countries suffer from HAI, whereas 15 in 100 patients in low-resource countries experience HAI (1, 2). In high-resource countries, the overall HAI prevalence is 7.6% (range: 3.5–12%) (3), while the data from low-resource countries is incomplete and fragmented; it consistently suggests significantly higher rates.

According to WHO's recent infection prevention report, more than 40% of hospitalized COVID-19 patients were HAI cases (4). As of 21 November 2022, there have been 633,522,052 confirmed cases with 6,599,100 associated deaths worldwide, according to WHO (5). There have been 2,036,416 confirmed cases with 29,421 deaths in Bangladesh (BD), 6,620,317 confirmed cases with 159,473 deaths in Indonesia (INA), and 26,654,729 confirmed with 30,111 deaths in South Korea (KR) reported to WHO (6–8). With a high number of patients seeking medical treatment combined with the limited hospital capacity during the COVID-19 pandemic, numerous studies have highlighted the impact of HAI caused to patients, healthcare workers, and the community (9–11). Available evidence shows significant HAI indicators increase in intensive care units (ICUs) in 7 low-middle-income countries during the COVID-19 pandemic (9). Centers for Disease Control and Prevention (CDC) also report significant increases in HAI of central line-associated bloodstream infections (CLABSIs), catheter-associated urinary tract infections (CAUTIs), and ventilator-associated events (VAEs) in US hospitals during COVID (12).

Also, the COVID-19 pandemic has highlighted the extent to which the importance of infection prevention and control (IPC) measures and the negative consequences of poor compliance with the recommended measures, such as the use of personal protective equipment (PPE) and hand hygiene (13–15). The amendments or new strategies were introduced and implemented to accommodate lessons learned from the studies (16–18). Healthcare workers (HCWs) remain a target population in those studies or recommendations, even from those countries where family carers play significant roles in patient care (15, 19–22). In many Asian countries, caring for a sick family member is a socially and culturally expected behavior. It is more ubiquitous than nurse-led care across Asia and sub-Saharan Africa, irrespective of resource availability (23). The definition of family carers or family caregivers in these Asian countries is not equivalent to the carers in Western settings as they engage in more physical care activities at patients' bedside in acute healthcare settings (24, 25).

Our previous study (25) examining IPC policies and guidelines across BD, INA, and KR also highlighted that regardless of the roles played at the point of care, HCWs were only considered at increased risk compared to the general population, such as family and private carers. There were no great differences across those three countries irrespective of resource availability regarding the recommended IPC behaviors. This may because the basis of IPC policies and guidelines from those countries are adapted from those global organizations or other Western countries such as the United States or Australia.

HCWs have been unanimously thought to be stakeholders in IPC strategies regardless of setting or country because of their role in patient care in Western healthcare settings. Modenese et al. (26) stated in their COVID-19 surveillance study exploring factors associated with COVID-19 infection risk among HCWs that HCWs, especially nurses and nurse aides are at increased risk of HAI because of their working tasks, which are closely associated with direct contact with patients. A similar claim was made by Abbas et al. (27) that HCWs are not only at increased risk but also play a role in amplifying the spread of infection due to the physical closeness during their work. However, in a similar vein, it could be hypothesized that family/private carers who deliver direct patient care may pose or be at the same risk as HCWs. In fact, our previous research (28) exploring the family involvement in care provision in hospitals across BD, INA, and KR reported that family members in those countries are highly involved in a broader range of care activities, including (1) invasive care activities (i.e., intravenous injection, feeding *via* nasogastric tube, suctioning, wound dressing); (2) body fluid exposure activities (i.e., changing incontinent pads, cleaning up urine, feces or vomit, assisting patient with urinal/bedpan, emptying a urine bag); (3) direct contact activities (i.e., changing the position of the patient, sponging, toileting, assisting with ambulation, applying a nebulised medication, getting dressing, feeding); and (4) patient zone contact activities (i.e., making a bed, washing linens and clothes, organizing meals and medication).

Because family involvement in patient care activities is a deeply embedded practice, family members or private carers have continued to carry out their roles as carers whilst staying next to their patients even under the strict implementation of IPC measures during the COVID-19 pandemic. Several reports cautiously reported the potential links of this care arrangement in hospitals to the spread of COVID-19 (10, 29–31). Despite the extensive involvement of family members in patient care and raised concerns about HAI, there have been no real-life experiences of patients and their carers about IPC practice. Understanding the context and the status of IPC practice at the point of care is fundamental to ensure IPC measures are effective. To date, very little is known about the risk of HAI transmission related to the involvement of family and private carers in the clinical setting and their participation and the level of engagement in recommended IPC behaviors, and the context of IPC promotion amongst patients and their family/private carers.

Thus, this study aimed to explore the context of IPC behaviors in which family carers/private carers provide direct care and to assess factors influencing family/private carers' compliance with recommended IPC measures across Bangladesh, Indonesia, and South Korea. The findings of this study are important to generate awareness and understanding of different landscape of care arrangement and IPC practices across many Asian countries and assist in developing functional IPC programs to protect all involved in the patient care.

## Materials and methods

### Design and study setting

We used a qualitative approach to understand the complex reality of care provided by family and private carers, and their engagement in recommended IPC measures across selected countries. With the social constructivism and pragmatism underpinning this study, the case study methodology was applied to obtain a deeper understanding of this phenomenon of care provision. According to Yin (32), the case study is “a*n empirical inquiry that investigates a contemporary phenomenon within its real-life context; when the boundaries between phenomenon and context are not evident; and in which multiple sources of evidence are used*”. As this study aimed to explore in depth the unexplored reality of family/private carers' involvement in inpatient care, the context of their engagement in IPC behaviors, and multiple individuals involved in the process, a case study methodology was deemed relevant. We used the Consolidated Criteria for Reporting Qualitative Research (COREQ) to ensure validity of methodological features.

The study was conducted at five tertiary level hospitals across three selected countries, namely Bangladesh (BD), Indonesia (INA), and South Korea (KR); two hospitals located in Rajshahi and Barishal of Bangladesh; two hospitals located in West Java province of Indonesia, and a hospital located in Gyeonggi-do province of South Korea. Data collection was done between July 2019 and February 2020. While most of the data collection was done prior to the emergence of COVID-19, two interviews were conducted during COVID-19.

Based on the literature review and consultation session with IPC experts and experienced qualitative researchers, the interview guides were separately developed for each role of participants, i.e., (1) patients and family/private carers, (2) healthcare workers. We chose a blend of structured and unstructured interviews to explore participants' perspectives deeper within the interests of the topic. Interview questions cover four key main areas: (1) knowledge and awareness of HAI and IPC strategies currently being implemented, (2) perceptions and attitudes toward IPC strategies or education, (3) experience around IPC recommendations or education, and (4) experience around care provision by family/private carers. It was then pilot tested in BD and KR to ensure the questions would elicit a response from participants within the interests of the topic and be understandable across three countries. The interview guides were modified based on the comments and feedback received. Interview guides are attached as a supplement.

### Trustworthiness/rigor

We used Lincoln and Guba's (33) evaluative criteria to ensure the trustworthiness of findings: credibility, dependability, transferability, and confirmability. We also sought assistance in the interpretation of these criteria from Creswell (34).

Several techniques were applied in our study to achieve trustworthiness during the qualitative inquiry. For credibility, amongst suggested techniques by Lincoln and Guba, we employed prolonged engagement, several triangulation methods, and peer debriefing throughout the data collection and analysis. Firstly, local researchers of BD and INA experienced in qualitative data collection had already established a rapport from multiple studies previously done at the sites. The first author who undertook the data collection in KR spent a prolonged time visiting the site every day during the data collection period to build trust with participants and observe various aspects of the setting. Secondly, data source and site triangulation were achieved by exploring different perspectives of multiple individuals, i.e., patients, family/private carers, and healthcare workers from multiple sites. Also, by being investigated by multiple researchers across the selected countries, researcher triangulation was attained. Researchers were constantly engaged throughout the study, including the preparation, data collection, and analysis phases.

For the dependability of findings to be repeatable, detailed descriptions of study methods were provided, including (1) the process of selection and recruitment of participants, (2) interview questions, (3) the process of interview, and (4) the process of analysis. For transferability, we applied purposive sampling using the eligibility criteria combined with convenience and snowball technique. For confirmability, which extends the findings could be confirmed by other researchers, we used direct quotes from participants when reporting the findings and applied several triangulation methods. Also, we engaged local external researchers to confirm the interpretation of IPC practice and the cultural context of the findings.

### Participants

Two groups of individuals were interviewed: (1) patients, their family and private carers; and (2) HCWs, including doctors, nurses, hospital managers and staff members.

When recruiting participants, we employed convenience, purposive, and snowball sampling techniques. To secure the sites across the selected countries, which could allow us to access the different groups of participants relevant to our study focus. Once the sites were confirmed, the criterion-based purposive sampling was applied. Eligibility criteria for participants are listed in Table 1. As addressed, the care provision by family carers in hospital setting is a normal phenomenon. Using the criteria, we chose participants based on the likelihood of illustrating phenomenon of care provision. Because of a rapport, the research teams across the three countries could join handovers and ward rounds to grasp the information about potential participants, and this assisted the selection of participants.

**Table 1:** Eligibility criteria for participants.

**Participant group**	**Requirements**
Patients:	■ Aged 18 years or over ■ Admitted to hospital for at least 24 hrs _ Had a family member or private carer during the hospital stay
Family members/Private carers:	■ Aged 18 years or over ■ Provided care for a patient for at least 5 h in a hospital
Healthcare workers:	■Had worked on the ward for at least 1 month ■ Provided clinical care to patients on a ward.

We also used the snowball technique as the existing participants invited other patients and their family carers in the same room or ward to the study. This process also occurred when recruiting HCWs for interviews.

Recruitment of participants continued until “the point in data collection and analysis when new information produces little or no change to the codebook” (35).

### Data collection

All interviews were conducted in the participants' native languages by researchers who were native speakers using the interview guides. Before commencing the discussions, an information sheet was provided, and informed written consent was obtained from all participants. Participants were informed how their privacy and personal information would be protected and gave their consent to the research. Interviews were conducted in various locations depending on the availability of venues and the preference of participants, for example, a patient's bedside, an empty patient room, an empty staff room, and a café in a lobby. The interviews varied between 17 and 55 min in duration, and all discussions were audio-recorded with the participant's agreement. Using the interview guides, research team members asked participants in open-ended manner to allow flexibility of sharing their thoughts and perspectives of the topic areas. Potential prompts for each key areas were discussed among the team members in the preparation phase. All interviews were transcribed verbatim in the local languages by the researchers who conducted the interviews. The transcripts were then translated into English professionally for those interviews collected from BD and INA. For the KR site, the first author, fluent in English and Korean, did the translation into English. The back-translation method was applied to ensure the accuracy of the translation for the first two transcripts by a qualified independent person. During the data collection, there was consistent engagement with the research team across three countries throughout the data collection phase to cross-check interim findings and ensure the interview process.

### Data analysis

Thematic analysis with an inductive approach was carried out to identify themes. NVivo12 was used for data management and coding. To find repeated patterns of meanings, the interviews were repeatedly listened to and took notes concerning infection prevention and control behaviors were. The first author, JYP, initially developed a list of initial codes, and then finalized the codes and themes in consultation with supervisors HS and JFP. The decided final themes and codes were discussed with other researchers and confirmed. Quotes were narrated to describe the themes and codes, but pseudonyms were used, when necessary, to protect the confidentiality and anonymity of participants. Consolidated criteria for reporting qualitative research (COREQ) were used to facilitate the research process (Supplemental material).

### Ethics

Ethical approvals were obtained from the University of New South Wales Human Research Ethics Committee (Approval No. HC 180919), International Center for Diarrhoeal Disease Research, Bangladesh (Approval No. PR-19012), Bandung Islamic University Health Research Ethics Committee (Approval No. 378/EC/I/19), and Catholic Medical College Institutional Review Board (VC19QEG0051).

## Results

We interviewed a total of 64 participants through 57 interviews, including 6 group interviews across three countries: 16 patients, 20 family carers, 1 private carer, and 27 healthcare workers.

Of the participants, 20 were from BD, 19 were from INA, and 25 were from KR. Of the 64 participants, 49 were women, and 37 were in group 1. Detailed characteristics of interview participants have previously been published (28).

The interview findings are subsequently presented based on the themes and subthemes that emerged from the data. A total of three main themes and eight subthemes are identified in Table 2.

**Table 2:** Themes and subthemes from the findings.

**Theme, subthemes**	**Description**
Theme 1	Assumptions
Subthemes	■ Levels of understanding and provision of training ■ Roles in the hospital
Theme 2	Knowledge of IPC and HAI
Subtheme	■ Understanding the transmission of HAIs ■ Linking knowledge to compliance with IPC ■ Knowledge vs. availability of IPC resources ■ Limited capacity to provide training
Theme 3	The role of culture
Subtheme	■ But it's my family member ■ Paying respect to family and friends is leading to overcrowded hospitals

### Assumptions

#### Levels of understanding and provision of training

During the interviews, the HCWs acknowledged that they made assumptions regarding the level of awareness and understanding of family and private carers toward clinical practice and IPC measures. The HCWs perceived that private carers would be trained and have the theoretical knowledge and practical skills to take care of a patient and perform appropriate infection control measures to carry out a range of clinical practices.

*Because the agency has already trained them (private carers) before they come here…we don't give any education to them but provide basic information about patients (KR, Nr, 003)*.

HCW felt private carers were more competent as they had past experiences, unlike family carers. It was suggested that only minimal educational support should be provided to private carers: “*because the private carers had their training…. Because the private carers are…experienced…but the family carers…we have to explain from scratch”. (KR, Nr, 005)*. However, the HCW acknowledged that they do not know the actual level of training or experience the private carer may have received or their competency in undertaking patient care activities.

If training is provided, one of the private carers explained that it was done at the bedside under the guidance of an experienced peer and mainly focused on manual handling and basic care skills: “*Similar to looking after a patient…basic theory …principles of caring…how to arrange a wheelchair and how to put a lock on the wheelchair… how to transfer a patient to a wheelchair... how to get a patient up in a standing position…position change…how to take a patient up by myself without help. Getting a patient dressed when a patient has an intravenous line…how to put an incontinent pad”. (KR, Pc, 001)*

#### Roles in the hospital

Although family and private carers provide a wide range of primary care activities to patients across those selected countries, there seemed to be no clear division of family carers' roles and nurses' roles in care activities.

*At first, I did not think this was what a family carer had to do…. Because this is my first time here, I did not know that this is a part of the job that a family carer needs to do…. Because it is not clear what the family carer has to do, and it is not communicated. So, is this what I should do, or is this for nurses to do? (KR, Fc, 007*)

It was suggested that neither nurses nor patients/family carers understood each other's role in the care activities, especially around basic care activities. Consequently, it was suggested that this lack of clarity led to negative implications for patient safety. A family carer caring for her daughter shared her experience of a near miss of her daughter's fall due to an unclear role in changing the patient's linen.

*I went to tell the nurses. I said, “there is much blood on the bed. I would like the bedsheet to be changed, please…...” The nurse answered “yes” when I asked for the sheet to be changed… But when we were about to leave the room, the nurse got the new sheet to us. …So, I changed the sheet while I got my daughter standing in the corridor. When I was changing it…I asked the nurse to help me. I just needed a little help, please. Then she helped me. After that, I found my daughter plopping down wearily on the floor….it could have been a significant incident…even other people passing by were startled…When I saw her like that, I got so angry…I thought all of these should be done by nurses for us. I was so upset it happened to my daughter while changing this………a part of it was my fault. I could have changed this when we returned from a walk…. (the patient's mother burst into tears) …more than that, I thought nurses should do this for us. (KR, Fc, 007*)

Some HCWs reported that the family carers did these jobs (i.e., changing bed linen) because of the heavy workloads of the hospital staff*: “it is not feasible for them (staff members) to do it for all patients, and we have other tasks to do so…we change it once or twice, but we usually ask family carers ….because it is not that difficult…so we teach them…so now, family carers or private carers are changing the bed linens….. I feel sorry to ask them to change bed linens....it is not anyone's task….it is not our job to do…actually, it is a nurse assistant's job…but there are too many patients to change them all…. I am sorry, but we ask family carers to do.” (KR, Nr, 004)*

A similar response was noted from a nurse from Korea about how the situation demands the family carers to step in.

*When we are busy, family members help us with suctioning, L-tube feeding… and suctioning and changing positions for patients are done mainly by family members. (KR, Nr, 001)*

*The nurse assistants empty the drain or other bags…. but if the bag is full, private carers occasionally open it and notify us. (KR, Nr, 007)*

The types of care activities that family or private carers were involved with varied across the interviews. In some settings, they are either done by nurses or family carers depending on the situation, such as medication administration, suctioning, L-tube feeding, etc... A participant (HCW) from Indonesia described how the medication administration was done between nurses and family carers depending on the situation:

*The carers also take care of the patient's medications. It is the nurses' duty, but sometimes the carers help. To help them eat, help them with their medications (INA, Nr, 002)*.

### Knowledge of IPC and HAI

#### Understanding the transmission of HAIs

From the interviews with patients and their carers, the aim of IPC practices was framed as preventing patients from acquiring HAIs; therefore, having a good understanding of IPC was important. However, during the interviews, it became apparent that the knowledge about HAI and IPC varied from very little to a reasonable level. Among those aware of the risk of infection, participants linked their concerns to issues with the environment and air quality and the risks associated with shared bathrooms and hospital equipment.

*Because she is not alone, she also shares the room with other people. And the patient next to her or around her, their illnesses are unknown to us, whether it is infectious or not... Especially when we share the bathroom…It is my main concern…… (INA, Fc, 005)*.

*I think it (HAI) is because of the air quality in the hospital environment…because the hospital is a place for sick people which is a potential for transmission of infections…. since the virus might transmit through air… (As an IPC measure, a family carer should) wear face masks….and she should not stay here too long (INA, Pt, 001)*.

Some participants linked the risk of infection to the actions of the patient or the family members, with concerns around the sharing of food and food utensils.

*The relationship between the two patients builds up so well (while staying in the hospital) that they don't even try to know about each other's disease. They even share their cups for drinking…drink tea from the same cup without knowing each other's condition. Thus, the infection spreads through that cup (BD, Nr, 005)*

*I have found some cases where the isolated patients shared food with their family members in the room…. their understanding of the risk might affect their behavior (INA, Nr, 003)*.

Interestingly, none of the family carers across the three countries acknowledged that they might pose an infection risk despite the length of time with patients and their involvement in the care provision.

#### Linking knowledge to compliance with IPC

Several participants (HCWs across all settings) claimed that the poor compliance to IPC measures among patients and their carers was because “*they did not know about it”*.

*Most of them (patients and their carers) did not take it seriously. They probably did not know the risks, or they simply did not know what the diseases would look like…. It is merely because they are unaware of the dangers (INA, Nr, 009)*.

Another nurse from INA claimed that the low IPC compliance among patients and their carers resulted from them not seeing the consequences of non-adherence.

*I think it has a connection. Because they simply do not know, they are clueless…because they are not used to washing hands and see nothing wrong with it. Some of the patients said that (INA, Nr, 010)*.

Similarly, a nurse from KR also stated that patients and their carers inconsistently comply with IPC measures because the implications of non-compliance to IPC measures were not apparent to them.

*Because the healthcare-associated infection is not something that ordinary people could feel…and it is not apparent or tangible….it may or may not happen…they (patients and their carers) vaguely think like that …and they don't feel it (KR, Nr, 008)*

Participants (HCWs) expressed their concerns that the lack of knowledge in practical components of hand hygiene (HH) practice among patients and their carers could be barriers to effective HH practice even though they performed HH. A nurse from INA reported that patients and their carers did not follow the recommended HH practice.

*There are steps to wash hands properly, yet they do not adhere to the steps…mostly just washing hands with water (INA, Nr, 003)*.

The lack of understanding of the source of infection was noted in the interview with a private carer from KR.

*I did not wear gloves when helping her with peeing because they did not look so dirty. And I can save some gloves by not wearing them…but I always wear gloves when handling feces…But when I am doing this (urinary catheter) …not wearing gloves, just grab and do it…...but my hands can get dirty when I am handling* feces…... *(KR, Pc, 001)*

#### Knowledge vs. availability of IPC resources

Many of the participants reported never using the alcohol-based hand rub (ABHR), with participants reflecting that the use of ABHR was a new practice to them, or that they were uncertain about what it was or whether they were allowed to use it, or that they just preferred to use soap and water. Some participants even went on to say that they did not trust the product.

*The hand sanitiser is always placed here, so if anyone needs it, they could use it…but I do not think people would use it…not many people use it here…we just wash hands with water when going to the bathroom (KR, Pt, 006)*.

*I did not know what (ABHR) was until now. Also, we are allowed to use it…Frankly speaking…. nurses use it occasionally. I only knew now that we could use it (KR, Fc, 010)*.

*We did not use this (ABHR) well…. I doubt whether this works or not (KR, Pt, 003)*.

Only after viewing the hospital staff using the ABHR did participants realize what it was for: “*Frankly speaking, I have not used it at all…in the morning, the professor who did the surgery for my daughter came for the round. And I saw him using this (ABHR) and his fellow doctors. Then I knew what this thing was for. I started using this since then. I learned by seeing it……I wondered why he was doing this (rubbing motion) …I thought this was a lotion.” (KR, Fc, 007)*

The availability of IPC resources was also noted as a significant hurdle to IPC compliance from BD and INA. For example, a nurse from BD *(BD, Nr, 004)* shared her frustration about the lack of sanitation facilities, i.e., water, basin, and soap:

*Many times, we can't do that (wash hands). It is true…. Even if I want to wash my hands with a bar of soap, sometimes it is impossible because of a shortage of materials (soap or handwash). Sometimes I faced a problem of water shortage even in the middle of washing my hands. So, we cleaned our hands with bottled water and a bucket. Is it possible to control infection this way? It is not possible*.

…*...We have a shortage of water as we have no basin…there is a basin for some patients (in the bathroom), but there is nothing like that in our ward…. nothing like this (ABHR) is provided to them (patients and their carers)*.

A patient from BD described what they do to deal with the situation: “*We bought everything…we bought masks, bought gloves every other day, we bought everything for washing hands…I do it from my expenses, not from the hospital” (BD, Pt, 001)*

Shortage of handwash soap and ABHR was also noted from INA sites. Patients and their carers from INA described the poor resource condition.

*No handwash soap. The water flows well, but there is no soap for handwashing. Soap bars are unhygienic as the germs might still be there….it (liquid soap) should be provided by the hospital and be placed at the sink over there…. there are none in here. There are some by the sink outside. But it is a hassle to come back and forth. For the most crucial thing, there should be one at the sink, for the most critical thing….and a towel (INA, Fc, 004)*.

Other participants from BD stated that people outnumbered by the available resources might contribute to IPC compliance and negatively affect HAIs.

*There are two bathrooms for the whole unit, and there are about two hundred patients….it has been seen that taps are broken from repeated use by patients…so there is no separate basin for handwashing…thus within the poor facilities, we try our best to take care of patients. There is no maximum use of limited resources here…here is...more than maximum utilization of limited resources (BD, Nr, 006)*.

*Both infected and non-infected patients stay in the same bed. If there are three patients (in the same bed), all of them are easily infected. Moreover, we have a scarcity of beds. For instance, eighty patients will have to accumulate in the 20 beds. How can they be separated? (BD, Dr, 008)*.

#### Limited capacity to provide training

Despite many healthcare workers' concerns around poor adherence to IPC measures among patients and their family carers, many reported they did not provide IPC education to patients and their carers. Workload issues were the primary reason for not being able to provide training to patients' family members or carers.

*It is difficult if there are 10–15 patients…. Because the private carers are…experienced…even though it may be minimum. So, we need to explain just a little. But the family carers are…they are like…we must explain from scratch. Totally from the basic…and if the family carer is changed, we must do it repeatedly (KR, Nr, 005)*.

### The role of culture

#### But it's my family member

Cultural norms around the value of maintaining physical contact with a sick family member seemed to influence participants' perception of IPC compliance. A family carer from BD who cared for her father-in-law gave her reasons why she chose not to use masks or gloves whilst caring for her family member.

*I know everything about this (IPC measures). But I don't wear masks or gloves. I don't take any of these. My father-in-law is not my blood-connected father. If I use it, he could think I am using it for his horrible smell…as I am his daughter-in-law. On the other hand, if I use gloves during his toilet, he could think that too. I don't want to let him feel like that. So, I don't use masks or gloves even though I know about that (BD, Fc, 006)*.

The participant also shared her previous experience of caring for her husband, who was admitted with TB:

*When my husband was a TB patient, I was informed to wear a mask when attending to my patient. They also told us to put a mask on my child. But we did not do that. Because he (husband) could become mentally weak by thinking that we hate him, people tell us that they hate the disease but not the patient…. for our close relatives, we can't do that (wearing gloves or masks)*.

A nurse from BD shared the same attitude toward precaution when caring for her family member. When asked if she had any experience of staying as a family carer and her experience of taking any precautionary measures, her responses were: “*I could not take proper precaution before my father as I am his daughter…...”(BD, Nr, 005)*

The participant explained the public perception of wearing PPE when caring for their family member.

*We tell them (family carers and patients) to do these (washing hands, wearing masks, gowning). Some patients do not wear masks, and neither patient's relatives. The patient feels bad if his relative uses masks while touching or being with him. When his close relatives are using masks whilst visiting him, he becomes terrified of thinking about what type of disease this is…. what might happen to him. We tell the attendant they must use the hand wash, though…we isolate the patient too (TB patient) (BD, Nr, 005)*.

The participant added her experience dealing with family carers who insisted on entering the insolation room to be with their patient diagnosed with TB.

*Visitors wanted to be in the ward…he asked why the door was locked and not this door…these types of questions…we needed to tell them many things to them convinced that the patient had TB. It may affect others if others stay in touch with him. Then, they said, “what? (asking with anger), it won't happen; you (nurse) are doing this to us, separating my patients to a different room, asking us to wear masks! No, we won't wear masks!” they showed anger like that. (BD, Nr, 005)*

The participant also described the case of a family carer who became sick whilst caring for a patient due to non-adherence to IPC measures.

*We had an attendant who got sick coming here to care for his patient…. even after cleaning the patient's feces or urine, and they sometimes did not wash hands properly…. he was doing this thinking ‘what would happen! The patient is my close relative, and he is my father, and my mother. The attendant thought he did not need to take any precautions for his relatives. (BD, Nr, 005)*.

#### Paying respect to family and friends is leading to overcrowded hospitals

Family members, close relatives, and friends visit patients in hospitals as an expression of closeness and respect. However, as outlined by the HCWs, it contributes to hospital overcrowding and has negative implications for patient care outcomes. A nurse from BD described the issue of having too many visitors around patients given IPC compliance.

*Visitors…there are challenges. We have to inform patients' visitors…when we go to a patient to give an injection, the syringe…I opened the syringe, but there were too many visitors, so I asked them to move, but they were not moving. If there was one visitor, we could ask them to stay aside. But when there were…ten people, they did not listen. If they remain close, they could touch the syringe or get poked by the needle. We often face many problems because of them (visitors). Besides, when there were ten visitors, it was impossible to provide masks to all of them. (BD, Nr, 005)*

Other participants also voiced their concerns about cross-infection due to an excessive number of patients and their accompanying family members and visitors.

*We have no visiting hours. Even though it is, it is on the paper. This is not implemented…. even though they were asked to leave, they entered the room again. Twenty-four hours except for night time, three to four people stay with each patient on average. So, this is the main obstacle to infection control (BD, Dr, 007)*.

Even though stricter IPC measures were in place to limit the number of visitors during the COVID pandemic, the customs of visiting patients seemed to continue among family members and relatives, according to a participant from KR.

*Due to COVID…many family carers are using their swipe card around among themselves. They should not do it, though... so we remind them again that only one person who stays with a patient can carry the card….so we are doing this. (KR, Nr, 008)*

## Discussion

Our study shows that assumptions about patients and their family/private carers, diverse levels of understanding, lack of IPC resources, and cultural values interdependently drive patients and their family carers to determine what action they would take when it comes to IPC practice during patient care. While previous studies (36–43) have linked issues around resources and knowledge to patient (and to some degree family member) compliance with IPC recommendations, our findings around the role of culture and its influence on engagement with recommendations are a unique contribution to the literature.

In our study, while the need to educate patients and their family members providing onsite care has been acknowledged, there are key barriers impacting implementation: (1) assumptions around the educational need and (2) capacity to deliver.

Across the three countries, assumptions are currently being made regarding the necessity of training/education being provided to carers. It appears that HCWs are potentially incorrectly assessing the level of awareness and skills that patients and the family/private carers have regarding IPC. These assumptions may be based on the past experiences that the HCWs have of working alongside private carers, or perhaps based on information received from the agencies that employ these private carers. Unfortunately, during our interviews, we did not sufficiently drill down into the HCWs decision-making processes to be able to state one way or another. However, it also does not explain why education or training is not being provided to patients' families. We believe that this issue may arise because there is no delineation in the roles and responsibilities between the family and private carers, and so there may be confusion about the need for training or education. If HCWs assume that family or private carers are only carrying out care activities assumed to be at low risk of potential exposure to a HAI, then it is not surprising that they are not providing any education.

Family carers/private carers are being placed in a position where they had to care for patients with limited knowledge of IPC measures required for their protection as well as the safety of their patients during patient care. The types of care activities being reported by these untrained family and private carers, have the potential to expose the carer or the patient to a HAI. This is more concerning for the private carers assigned to patients requiring complex care activities based on the common assumptions that private carers were trained and experienced.

Due to the high patient to nurse ratio, it was not surprising that the nurses we interviewed spoke about having limited time to engage with the family/private carers. Numerous studies have linked heavy workloads with negative impacts on patient safety, including an increased rate of HAI (44–46). Not only does it impact on the quality care provided to patients but also negatively impacts on nurse-patient communication and their attitude and motivation toward IPC education. It also means that it is not appropriate to suggest that the responsibilities for support the IPC understanding and skills development of family or private carers should be placed on the shoulders of these over-stretched staff members. Calls have been made by the WHO and other organizations to governments to improve the workforce opportunities and career pathways of staff members dedicated to IPC. With funding and support, it may be that the integration of patient and family member engagement strategies around IPC could fall within the responsibilities of dedicated IPC professionals within clinical settings. Alternative options include volunteers within the clinical systems, such as retired nurses or other healthcare providers who are trained up to be patient advocates and IPC ambassadors and fulfill the role of support family and private carers to ensure their safety during their time supporting the patients.

Obviously, aside from the issues already raised, we acknowledge that without the adequate resources within these clinical environments, there will always be limitations to patients and their carer's engaging in relevant IPC activities. A study exploring HH infrastructure in Bangladesh (12) observed that family carers had more than double HH opportunities compared to HCWs. Still, their compliance rate was lower than HCWs. The authors reported that fewer available HH resources, such as no available basin with running water and no available hand hygiene materials for patients and their carers, significantly influenced HH compliance, amplified by their lack of knowledge around IPC measures. This is consistent with our study findings, which showed a lack of basic infrastructure for HH, i.e., water, basin, and soap, from Bangladesh and Indonesia sites. Previous studies (41, 47, 48) on HH compliance have consistently suggested there is a need for enhanced provision of HH facilities and materials.

Within each of the countries that were included in the study, there was a sense of “*deep culture”* associated with the values placed on caring for family members (49). As Ogbu (50) noted, this unique care arrangement has been shaped by cultural tasks that people in the culture have come up with to solve common problems in life as solutions over a long period. If we are going to support change moving forward, it is critical that we consider these cultural beliefs and values associated with caring activities. For example, family member may not willingly comply with the use of PPE, if they feel that this could be construed as rude or disrespectful to the patient who is their family member, or that it could be a physical barrier stopping them from expressing their connectiveness to the family member. Previous studies investigated the outbreaks of Ebola and Nipah and IPC compliance, noted the influence of social norms and how people prioritize physical contact with sick families over the recommended IPC measures (51–54). The need for culturally responsive IPC strategies that consider these social norms and the influence they may have on compliance with recommendations is critical. As a starting point, government and other agencies must work with patients and their family members to co-design guidelines, education packages and communication materials which account for the local practices. This is not only relevant to the countries that have been included in this study, but also other country settings with high levels of migration.

This study has two limitations. Firstly, we could only recruit one private carer across three countries due to the unavailability of private carers during the data collection period. Having a single participant of private carer from one study site amongst three countries may not truly reflect the perspective of the participant group. It may affect the internal validity of the study. Secondly, although the study included multiple participants, including patients, family carers, private carers, and healthcare workers, no doctor was recruited from a Korean site. This was because the data collection period overlapped with the change-over period for ward residents.

Nevertheless, this study has several strengths. To the best of our understanding, this is the first study to qualitatively explore the issue about family/private carers IPC compliance. Our study acknowledged the real-life context of IPC behaviors and compliance among family/private carers and explored the factors that truly matter to patients and their carers when adhering to the recommended IPC measures using in-depth interviews, irrespective of resource settings across Asia. The findings from this study will inform policy makers and public health organizers to update future IPC programs and to develop adequate and practical strategies and recommendations that embracing all involved in the patient care.

## Conclusion

Improving the understanding and skills of patients and their family carers to engage in relevant IPC strategies needs to be addressed, especially given the current pandemic, where there is growing dependence on family caregivers' involvement in the care provision (55–57). This study has provided an in-depth understanding of factors influencing IPC compliance among patients and their family carers across three countries. This study also suggests future research for more qualitative investigation throughout the rest of Asia and sub-Saharan Africa and to explore whether the factors differ. A multifaceted approach should be developed collectively with a broader range of experts, including validation of cultural influence and its influence over healthcare arrangements.

## Data availability statement

The raw data supporting the conclusions of this article will be made available by the authors, without undue reservation.

## Ethics statement

The studies involving human participants were reviewed and approved by ethical approvals were obtained from the University of New South Wales Human Research Ethics Committee (Approval No. HC 180919), International Center for Diarrhoeal Disease Research, Bangladesh (Approval No. PR-19012), Bandung Islamic University Health Research Ethics Committee (Approval No. 378/EC/I/19), and Catholic Medical College Institutional Review Board (VC19QEG0051). The patients/participants provided their written informed consent to participate in this study.

## Author contributions

JYP led the study across three countries. JYP led, JFP and HS supported study methodology. MSI and KC led the project administration in Bangladesh (BD), TR and EN led the project administration in Indonesia (INA), and JYP led the project administration in South Korea (KR). JYP for KR, KC for BD, TR and EN for INA managed and conducted the data collection. JYP led formal analysis assisted by HS and JFP. All authors discussed and contributed to the decided final themes. JYP wrote the main manuscript, and HS and JFP supported subsequent revisions. All authors approved the final version of the manuscript.
