# Supplementary material for: Exploring factors influencing the compliance of patients and family carers with infection prevention and control recommendations across Bangladesh, Indonesia, and South Korea
Source: Front Public Health. 2022 Dec 22;10:1056610. doi: 10.3389/fpubh.2022.1056610 (PMC9815766; doi:10.3389/fpubh.2022.1056610)
Supplement: Supplementary file 2 [file Data_Sheet_1.pdf]

Interview Preparation

1. Explain the participant information sheet, and have Interview face sheet ready
2. Consent forms explained and signed (please include permission to use an audio-recording device during the interview)
3. Keep the key areas in. Take notes during the interview to share thoughts

Key areas to explore

1) Participant's knowledge and awareness of healthcare-associated infection and IPC strategies being implemented currently
2) Perceptions and attitudes towards IPC strategies/education,
3) Experiences around IPC education/measures (difficulties, feelings, previous experience)
4) Experiences around care provision by family carers/private carers

Interview face sheet

What is your gender? ☐ Female ☐ Male

Please indicate your age range?
- ☐ ≤24 years
- ☐ 25-34 years
- ☐ 35-44 years
- ☐ 45-54 years
- ☐ 55-64 years
- ☐ 65 years

What is your highest qualification? (Please tick one box)
- ☐ No formal schooling
- ☐ Less than primary school
- ☐ Primary school completed
- ☐ Secondary school completed
- ☐ High school (or equivalent) completed
- ☐ College/ Pre-university completed
- ☐ University completed
- ☐ Postgraduate degree completed

The below is only applicable to healthcare workers and private carers

What is your current role:___________________________________

How many years have you been working in field:_____________(years)
How many years have you been working in your current position: _____________(years)

In-depth interview guides

Guide for healthcare workers

Please tell me your position and how long you have been working here for?

Opening the interviews.

Acknowledge their answers and articulate the answers the interviewee gives.

E.g., You have seen many changes in IPC.

Please do not feed them information

If the answers are too broad, re-direct them to the study aims.

Based on their answer, you may use, "you have worked here long…years, and you have probably seen many changes in the hospital/ward in terms of infection prevention control." then move to the next question about strategies.

Could you describe the strategies used in this hospital to prevent the transmission of healthcare-associated infections to patients?

Prompts can be given about the standard precaution, including hand hygiene, use of personal protective equipment, healthcare waste segregation, transmission-based precautions, patient placement, etc.

In your opinion, how effective are these strategies? What factors do you think impact the success of these strategies?

Articulate more by asking about ward level, facility level

Whose responsibility is it to prevent opportunistic infection in a hospital? Why?

If a patient or family members of patients were not brought up, maybe prompt by asking, "What about patients/their family members?" "Should they have a role?"

Can you describe the role that patients' families/or private carer's have in providing care to the patient?

Prompt includes feeding, bathing, dressing, toileting, and administering medications.

Do you think that these activities pose a safety risk?

Wait for the responses without prompts.

If nothing is said, then prompt infection risks

What experiences have you had around recommending the IPC strategies (probs: hand hygiene) to patients/family members/carers?

Why did you recommend those (from their answer)? Was it a policy guide you to do, or did you see the risk associated?

In your experience, do patients comply with hand hygiene? What about their family members who are providing care? How would you feel if you saw someone is not compliant with recommendations for infection control strategies?

Do you think we should educate patients and family members/carers about infection prevention and control? What info would be relevant?

What method do you think is the best to deliver the information on infection prevention and control to patients and family members/carers in the hospital? Why?

What method do you think patients and family members would engage more in the infection program? Are there any suggestions you would like to make in terms of improving infection prevention and control at this hospital?

Closing

Thank you for participating in the interview. We have talked about [summary of main points of interview]. Is there anything you would like to add?

I would like to reassure you once again about the confidentiality of your information. As mentioned earlier, you can withdraw at any time. Feel free to contact me about your decision.

Guide for patients

Can you tell me why you are hospitalised and how long you have been admitted?

What has been your experience of staying in this hospital?

What type(s) of safety problem(s) do you think can occur in the hospital

> If answers are too broad, direct them to the study aim. And prompt for infection if not mentioned.
>
> "Among those concerns patients may have for the hospital stay, we are particularly interested in the healthcare-associated infection. Did you have any concerns about potential infection in the hospital?"

Do you know anyone who has experienced a safety problem at the hospital?

> Prompt for infection if not mentioned.
>
> Prompt for a personal experience if not mentioned

Do you know anything about why people may experience infections whilst they are in the hospital?

Are you aware of any infection risks for yourself associated with this hospital stay?

Have you received any information about infection prevention and control during your stay? If so, where did you get the information from?

Who is looking after you here? What kinds of caring activities are your family members/friends/private carers providing to you?

Do you have many visitors? Did your visitors help you with any care while they were visiting you?

If so, did they use any PPE? Did they wash their hands?

Did your carer receive any information or education about the roles they have to do as a carer in the hospital? If so, where did they get the information from?

> Articulate whether the information includes infection prevention and control measures.

Do you think there are any risks associated with being cared for by a family member?

What strategies do you think can be used to prevent and control this infection in the hospital?

(Prompts: Hand hygiene, PPEs-gloves, masks)

Whose responsibility is it to prevent opportunistic infection in a hospital? Why?

What do you see as your role in achieving good outcomes for you from this admission? What about your family members that come and care for you?

How would you feel if your caring person is not compliant with recommendations for infection control strategies? What action would you take if you encounter your caring person does not do what is recommended to?

What roles do you think you could play if you are invited to prevent hospital-acquired infection/nosocomial infection?

Do you feel that you need any more information about healthcare-associated infections or about strategies that you or your family could be involved with?

Are there any suggestions you would like to make in terms of improving infection prevention and control at this hospital?

Closing

Thank you for participating in the interview. We have talked about [summary of main points of interview]. Is there anything you would like to add?

I would like to reassure you once again about the confidentiality of your information. As mentioned earlier, you can withdraw at any time. Feel free to contact me about your decision.

Guide for family members and private carers

Can you tell me whom you are caring for? How are you related to the patient? Could you tell me why the patient is hospitalized for?

How long you have been caring for a patient in a hospital? How much time do you spend with the patient?

You could specify by hours per day, days per week.

Could you tell me about the care activities you are providing to the patient? Did you receive any training in caring for the hospitalised patient prior to performing the caring role?

Does your sick family member have many visitors? Did visitors help you with helping a patient while they were visiting? (or did visitors help a patient with any care while they were here?)

If so, did they use any PPE? Did they wash their hands?

What type(s) of safety problem(s) do you think can occur in the hospital?

Prompt: do you have any concerns about your family member's safety in a hospital?

Do you have any concerns about your own safety while in the hospital?

Do you know anything about how or why people may experience infections whilst they are in the hospital?

Are you aware of any infection risks for the patient you are looking after associated with this hospital stay?

What strategies do you think can be used to prevent and control this infection in the hospital?

(Prompts: Hand hygiene, PPEs-gloves, masks)

Whose responsibility is it to prevent opportunistic infection in a hospital? Do you think that family members can be involved with prevention strategies?

Have you received any information about infection prevention and control during your stay? If so, where did you get the information from?

Would you be willing to be involved with infection prevention and control programs? What do you think could be your role? What would support you to be involved?

Closing

Thank you for participating in the interview. We have talked about [summary of main points of interview]. Is there anything you would like to add?

I would like to reassure you once again about the confidentiality of your information. As mentioned earlier, you can withdraw at any time. Feel free to contact me about your decision.
